# Mapping MKP-3/FOXO1 Interaction and Evaluating the Effect on Gluconeogenesis

**DOI:** 10.1371/journal.pone.0041168

**Published:** 2012-07-25

**Authors:** Ping Jiao, Bin Feng, Haiyan Xu

**Affiliations:** 1 Hallett Center for Diabetes and Endocrinology, Rhode Island Hospital, Alpert Medical School of Brown University, Providence, Rhode Island, United States of America; 2 School of Pharmaceutical Sciences, Jilin University, Changchun, Jilin Province, China; University of Pecs Medical School, Hungary

## Abstract

**Background:**

MAP kinase phosphatase 3 (MKP-3) is known to attenuate the ERK signaling pathway. It has been recently demonstrated that MKP-3 is also a player in promoting hepatic glucose output in obese state by interacting and activating FOXO1. Reduction of hepatic MKP-3 expression is sufficient to reduce blood glucose levels in both diet-induced and genetically obese mice.

**Methodology/Principal Findings:**

In current study, the mechanism of MKP-3/FOXO1 interaction and the effects on transcription of gluconeogenic gene and glucose output was investigated in Fao hepatoma cells by using mutated MKP-3 and FOXO1 adenoviral constructs. The results indicate that MKP-3 phosphatase activity is not required for MKP-3/FOXO1 interaction but is essential for FOXO1 nuclear translocation and MKP-3 promoted gluconeogenesis. Compared to GFP control (1±0.38), MKP-3 increased G6Pase gene expression by 242% (3.42±0.62) while inactive MKP-3 does not change G6Pase expression (0.98±0.17). The residues 200–260 of MKP-3 and the residues 360–456 of FOXO1 are essential for mediating MKP-3/FOXO1 interaction. Interestingly, ERK phosphorylation deficient but not Akt phosphorylation deficient FOXO1 mutant lost interaction with MKP-3. Furthermore, in vivo experiments showed that Akt phosphorylation resistant FOXO1 3A mutant is sufficient to rescue the hypoglycemia caused by MKP-3 knock down in the liver of lean mice (from 141±6.78 to 209±14.64 mg/dL).

**Conclusions/Significance:**

1) Critical residues mediating MKP-3/FOXO1 interaction have been identified; 2) ERK phosphorylation deficient FOXO1 mutant is as potent as Akt phosphorylation deficient FOXO1 mutant in activating transcription of gluconeogenic genes; 3) Constitutively active FOXO1 can rescue the hypoglycemic effect caused by reduced hepatic MKP-3 expression in vivo.

## Introduction

Obesity is becoming a public health burden and poses as a risk factor for development of many life threatening diseases, particularly insulin resistance and type 2 diabetes [1], [2]. Impaired insulin action in obese state is reflected by increased glucose output from the liver and decreased glucose utilization by adipose tissue and muscle [3], [4], [5]. Dysregulation of hepatic glucose homeostasis in obesity is mainly caused by increased gluconeogenesis. Suppression of hepatic glucose output has been shown to be an effective therapeutic approach for controlling hyperglycemia in type 2 diabetes. Insulin is an important hormone for suppressing liver gluconeogenesis mainly through Akt mediated phosphorylation and inactivation of FOXO1, a transcription factor that stimulates expression of gluconeogenic genes by directly binding to the promoters [6], [7], [8], [9]. In addition, FOXO1 is also reported to increase transcription of PPARγ coactivator 1α (PGC1α), which plays an important role in amplifying gluconeogenesis [10], [11]. Besides Akt-mediated phosphorylation of FOXO1, insulin can also suppresses gluconeogenesis through disruption of FOXO1/PGC1α interaction [12]. Recently, insulin has also been found to repress gluconeogenic gene expression through Akt mediated phosphorylation and activation of salt inducible kinase 2, which subsequentially phosphorylates, inactivates and promotes degradation of the cAMP responsive coactivator TORC2 [13], [14]. It is worthy to note that insulin is not the only hormone suppressing gluconeogenesis, other hormones such as leptin and adiponectin, have also been reported to suppress gluconeogenesis [15], [16].

Tremendous efforts have been directed to study the mechanism of obesity-related insulin resistance. Our recent efforts for identifying novel genes that antagonize the effect of insulin on suppressing transcription of the *pepck* promoter, a key gluconeogenic enzyme controlling the first step of gluconeogenesis, revealed a previously unrecognized role for MAP kinase phosphatase 3 (MKP-3) in promoting hepatic glucose output. Hepatic MKP-3 expression is significantly increased in both diet induced obese and genetically obese rodents. In vivo loss of function studies through RNA interference in lean, genetically obese and diet induced obese mice confirms the role of MKP-3 in glycemic control [17], [18].

MKP-3 belongs to the family of dual specificity phosphatases, which contain eleven known members up to date [19], [20], [21]. These dual specificity phosphatases play an important role in attenuating MAP kinase signaling and they have distinct tissue distribution patterns and substrate preferences [22]. MKP-3 is recognized as a highly specific phosphatase for attenuating ERK1/2 signaling and enhanced basal ERK1/2 phosphorylation has been observed in the heart of MKP-3 deficient mice [23], [24], [25]. Existing literature indicate that ERK is not required for mediating the effect of insulin on suppressing gluconeogenesis though ERK can suppress expression of glucose-6-phosphatase, the key enzyme responsible for the last step of gluconeogenesis, independent of insulin action [26], [27], [28]. The unexpected cross talk between MKP-3 and the metabolic signaling arm of insulin action on controlling glucose output led to identification of FOXO1 as a novel MKP-3 substrate [18]. MKP-3 has been shown to interact with and dephosphorylate FOXO1, thereby promote its nuclear translocation, which is an essential step for turning on the gluconeogenic program through initiation transcription of gluconeogenic genes [18].

In this study, the mechanism of MKP-3/FOXO1 interaction and the effect on transcription of gluconeogenic genes and glucose output has been investigated in Fao hepatoma cells. Furthermore, the effect of the Akt phosphorylation resistant FOXO1 mutant on rescuing the hypoglycemia in mice with reduced hepatic MKP-3 expression was also studied.

## Methods

### Ethics statement

Animal experiments were approved by the Institutional Animal Care and Use Committee of Rhode Island Hospital (protocol 0093-11).

### Reagents and cells

Flag-FOXO1, Flag-FOXO1 3A, and Flag-FOXO1 9A expression plasmids were provided by Dr. Akiyoshi Fukamizu (University of Tsukuba, Japan). Ad-Flag-FOXO1 was provided by Dr. Pere Puigserver (Dana Farber Cancer Institute, Boston, MA, USA). PEPCK-Luc was provided by Dr. Daryl Granner (Vanderbilt University, TN, USA). G6Pase-Luc was provided by Dr. Richard M. O'Brien (Vanderbilt University, TN, USA). GFP-hFOXO1 was purchased from Addgene (Cambridge, MA, USA) [29]. Dexamethasone, sodium pyruvate and sodium lactate were purchased from Sigma (St Louis, MO, USA). Antibodies for MKP-3, Flag, and anti-goat IgG-HRP were purchased from Santa Cruz Biotechnology (Santa Cruz, CA, USA). ERK, phospho-ERK, FOXO1, pSer256-FOXO1, anti-mouse and anti-rabbit IgG-HRP antibodies were purchased from Cell signaling (Danvers, MA, USA). Tubulin antibody was purchased from Abcam (Cambridge, MA, USA). HEK 293A cells were purchased from Invitrogen (Life Technologies, Carlsbad, CA, USA). Fao cells were provided by Dr. Zhidan Wu (Novartis Institutes for Biomedical Research, Cambridge, MA, USA) [17].

### Construction of adenoviruses expressing MKP-3 and FOXO1 mutants

Adenovirus expressing MKP-3 and shMKP-3 were constructed in our previous study (16). MKP-3 C293S and MKP-3 S159AS197A were constructed by site-directed mutagenesis in plasmids. The coding sequences of MKP-3 153-381, MKP-3 C293S, MKP-3 S159AS197A, MKP-3 1–200, MKP-3 1–260, MKP-3 1–320, FOXO1 3A, FOXO1 9A, FOXO1 1–280, FOXO1 1–360, FOXO1 1–456 and FOXO1 1–558 were amplified by PCR, cloned into the entry vector and sequence confirmed. Then the above mentioned coding sequences were recombined into the Gateway-based pAd-CMV DEST^TM^ vector (Life Technologies) according to the manufacturer's instructions. Amplification of recombinant adenovirus was performed according to the manufacturer's instructions using HEK 293A cells. Large-scale amplification and purification of recombinant adenoviruses were performed using the ViraBind^TM^ Adenovirus Purification Kit according to the manufacturer's instructions (Cell Biolabs Inc., San Diego, CA, USA).

### Transfection of Fao cells and luciferase assay

Fao cells were transfected with Fugene HD reagent (Roche Diagnostics, Indianapolis, IN, USA) upon 50% confluency. The DNA: Fugene HD ratio was 1∶2.5. The Renilla luciferase expression plasmid was co-transfected as an internal control. Twenty-four hours after transfection, Fao cells were incubated overnight in serum-free RPMI 1640 medium. Forty-eight hours after transfection, cells were rinsed once in PBS and lysed in passive lysis buffer by two freeze-thaw cycles. Luciferase assay was performed using the Dual-Glo luciferase assay kit from Promega (Madison, WI, USA). The results were expressed as relative luciferase activity by normalizing firefly luciferase activity to renilla luciferase activity.

### RNA extraction and Real-time PCR analysis

RNA samples were extracted using the TRIZOL® reagent from Invitrogen according to the manufacturer's manual. For real-time PCR analysis, random hexamers were used for reverse transcription. After reverse transcription, real-time PCR analysis was performed in a 15 μl reaction in 96-well clear plates using Power SYBR® Green RT-PCR Reagents Kit on an ABI thermal cycler Step-One Plus (Life Technologies). Reactions contained 1x reaction mix, 5.5 mM MgSO_4_, 300 nM forward primer, 300 nM reverse primer, and SYBR green. PCR conditions were: 50°C for 2 min followed by 95°C for 10 min for 1 cycle, and then 95°C for 15 sec followed by 60°C for 1 min for 40 cycles. 28S rRNA or β-actin was used as reference. The sequences of the primers are as the following:

28S forward, TTCACCAAGCGTTGGATTGTT;

28S reverse, TGTCTGAACCTGCGGTTCCT;

G6Pase forward, CCCAGACTAGAGATCCTGACAGAAT;

G6pase reverse, GCACAACGCTCTTTTCTTTTACC;

PEPCK forward, CTGCATAACGGTCTGGACTTC;

PEPCK reverse, CAGCAACTGCCCGTACTCC;

MKP-3 forward, ATAGATACGCTCAGACCCGTG;

MKP-3 reverse, ATCAGCAGAAGCCGTTCGTT;

PGC-1α forward, TATGGAGTGACATAGAGTGTGCT;

PGC-1α reverse, CCACTTCAATCCACCCAGAAAG;

β-actin forward, CCAGTTGGTAACAATGCCATGT;

β-actin reverse, GGCTGTATTCCCCTCCATCG

### Glucose output assay

Fao cells were seeded on 12-well plates at 1 million/well in RPMI 1640 supplemented with 10% FBS. The next day, cells were transduced with adenoviruses expressing GFP, MKP-3, MKP-3 153–381, MKP-3 C293S or MKP-3 S159AS197A. Forty-eight hours post-transduction, cells were incubated in serum-free RPMI 1640 medium containing 1 μM dexamethasone for 5 hr. Cells were then incubated in serum-free, phenol red-free, glucose-free DMEM containing 1 μM dexamethasone, 2 mM pyruvate and 20 mM lactate. Supernatants were collected 3 hr later and subjected to glucose measurement using the Amplex® Red Glucose Assay Kit (Life Technologies). Cells were lysed and protein concentration was determined for each lysate sample. The glucose output rate was normalized by cellular protein content.

### Immunoprecipitation and western-blot analysis

To prepare cell lysates, Fao cells were washed with ice-cold PBS once and lysed with lysis buffer supplemented with protease inhibitors. To prepare liver lysates, livers were immediately frozen in liquid nitrogen, pulverized into powder and approximately 100 mg were used for homogenation in lysis buffer supplemented with protease inhibitors. To immunoprecipitate MKP-3 from cell or liver lysates, thirty microliters of Exactra D immunoprecipitation matrix (Santa Cruz Biotechnology) slurry were used to preclear cell or liver lysates at 4°C for 30 min. Then forty microliters of MKP-3 antibody-bound Exactra D immunoprecipitation matrix slurry were added to pull down MKP-3. For direct western analysis, one hundred micrograms of cell or liver lysate from each sample were used. Following PAGE on 4–12% gel (Bio-Rad Laboratories, Hercules, CA, USA), the resolved proteins were transferred onto PVDF membranes. Membranes were blocked in 1% BSA/1xTBST or 5% milk/1xTBST for 1hr followed by incubation with the appropriate primary antibodies in the presence of 1% BSA/1xTBST or 5% milk/1xTBST. Finally, membranes were incubated with appropriate horseradish peroxidase-linked secondary antibodies for 1hr in 5% milk/1x TBST. Protein bands were detected by ECL western blotting detection reagent (Perkin Elmer, Waltham, MA, USA) and images were taken on the Alpha-Inotech fluorochem image system.

### FOXO1 localization

Fao cells were infected with adenoviruses expressing a control protein (an inactive kinase) or MKP-3 or MKP-3 mutants. Twenty-four hours post infection, cells were transfected with GFP-FOXO1 expression plasmid. Forty-eight hours after infection, cells were incubated in serum-free RPMI 1640 medium in the presence of Veh or 1 μM Dexamethasone (Dex) overnight. Next day, cells were incubated in serum-free and glucose-free DMEM medium supplemented with 2 mM sodium pyruvate and 20 mM sodium lactate for another three hours in the presence of vehicle or Dex. Cells were then fixed in 5% PBS-buffered formalin for 10 minutes and examined for FOXO1 localization. Nuclear localization was quantified by counting cells based on predominant nuclear localization vs total number of cells.

### Mouse models and administration of adenoviruses

Male C57BL/6 mice were purchased from the Jackson Laboratory (Bar Harbor, ME, USA) at the age of 10 weeks. After one week of acclimation, mice were randomized into experimental groups with equal body weight and fed blood glucose levels. Each mouse was injected with 1×10^11^ viral particles expressing GFP or FOXO1 3A plus 9×10^11^ viral particles expressing shGFP or shMKP-3. Six days post injection, fed body weight and blood glucose levels were measured. Then mice were fasted overnight. Fasting body weight and blood glucose levels were measured seven days post injection, followed by sacrifice for tissue and plasma collection.

## Results

### Effect of ERK binding domain and phosphatase activity on MKP-3 promoted gluconeogenesis

**Figure 1:**
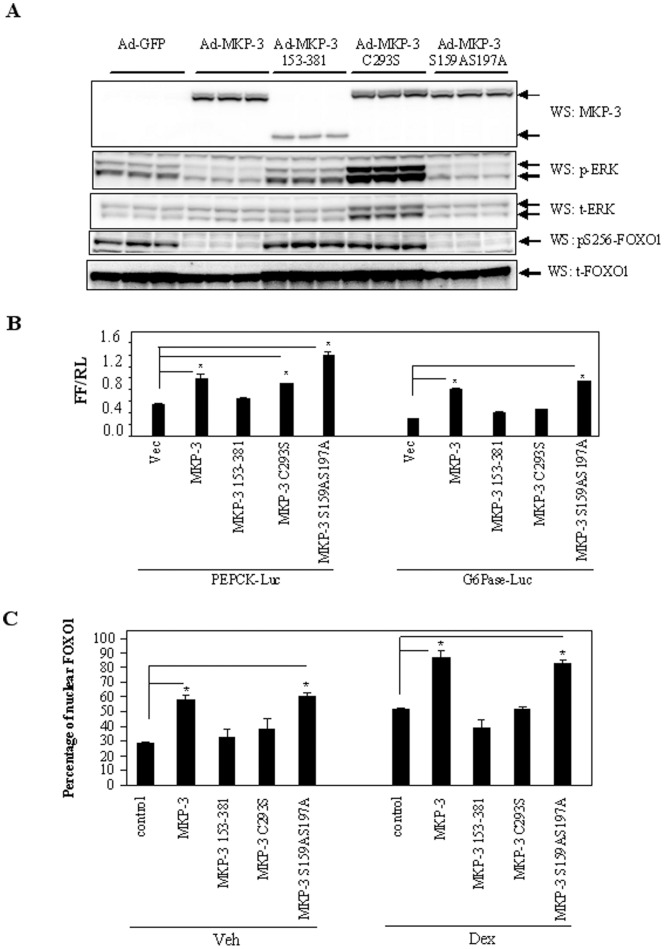
Effect of ERK binding domain and phosphatase activity on MKP-3 promoted transcription of gluconeogenic genes and FOXO1 nuclear translocation. **A.** Overexpression of MKP-3 WT or mutants deficient in ERK binding domain, deficient in phosphatase activity or deficient in ERK phosphorylation sites in Fao cells and the effect on phosphorylation of ERK and FOXO1. MKP-3 WT or mutants were over-expressed in Fao cells via adenovirus-mediated gene transfer. **B.** Effect of MKP-3 wild type (WT) or mutants on transcription of PEPCK and G6Pase promoters in Fao cells. Results were presented as ratios of firefly luciferase activities (FF) versus renilla luciferase activities (RL). **C.** Effect of MKP-3 WT or mutants on nuclear translocation of FOXO1-GFP in Fao cells. Vec, vector; Luc, luciferase; veh, vehicle; Dex, dexamethasone. *, P<0.05, MKP-3 wild type or mutant expressing cells versus control cells expressing an empty vector (B) or an inactive kinase (C).

**Figure 2:**
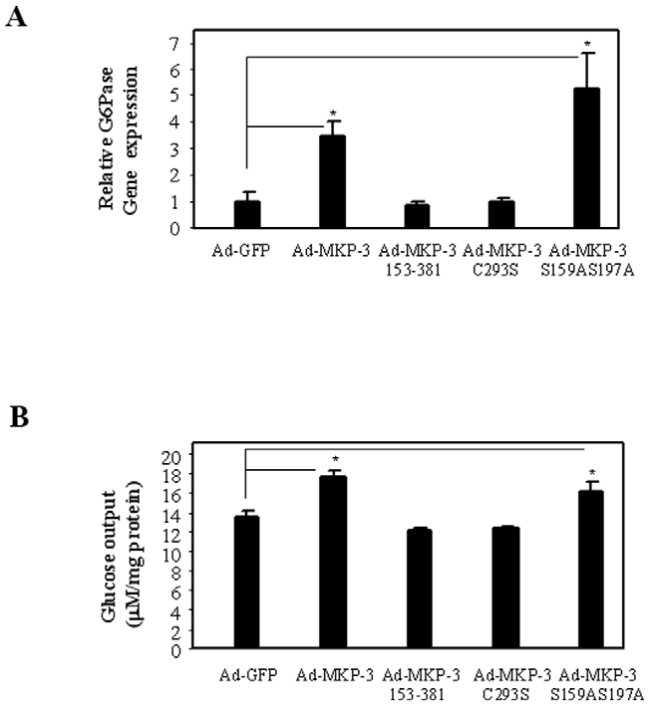
Effect of MKP-3 ERK binding domain and phosphatase activity on expression of endogenous G6Pase gene and glucose output in Fao cells. **A.** Over-expression of MKP-3 WT or mutants in Fao cells and the effect on expression of endogenous G6Pase gene. **B.** Overexpression of MKP-3 WT or mutants in Fao cells and the effect on glucose output. *, P<0.05, MKP-3 wild type or mutant expressing cells versus control cells expressing GFP.

To dissect the mechanism of MKP-3 promoted gluconeogenesis and MKP-3/FOXO1 interaction, three MKP-3 mutants were generated: 1) MKP-3 153–381, a N-terminal truncated mutant lacking the ERK-binding domain (mouse MKP-3 contains 381 residues); 2) MKP-3 C293S, an inactive mutant with the critical residue in the catalytic core of MKP-3, cystein 293, mutated to serine; 3) MKP-3 S159AS197A, a mutant deficient in two ERK phosphorylation sites which has been reported to resist serum induced MKP-3 protein degradation. Western-blot analysis indicated that all mutated MKP-3 proteins were properly expressed in Fao cells (Figure 1A, the top panel). To examine the phosphatase activity of MKP-3 and mutated MKP-3, phospho and total ERK (referred as pERK and tERK hereafter, respectively) levels were examined. MKP-3 reduced pERK level compared to GFP control (Figure 1A, the middle two panels). The phosphatase activity was significantly impaired in MKP-3 153–381 as reflected by no reduction of pERK level in comparison to GFP (Figure 1A, the middle two panels). Interestingly, MKP-3 C293S behaved like a dominant-negative mutant by suppressing the function of endogenous MKP-3 and caused an increase of ERK protein and ERK hyper-phosphorylation (Figure 1A, the middle two panels). In contrast, MKP-3 S159AS197A behaved like MKP-3 WT and decreased the level of pERK. In addition, phosphorylation levels on Ser256 of FOXO1 were also used to examine activities of MKP-3 since we previously reported that the insulin antagonizing effect of MKP-3 on gluconeogenesis was at least partially attributed to dephosphorylation of FOXO1 on Ser256. MKP-3 wild type and MKP-3 S159AS197A effectively prevented insulin stimulated phosphorylation on Ser256 of FOXO1 while MKP-3 153–381 and MKP-3 C293S did not have any effect on dephosphorylating Ser256 (Figure 1A, the bottom two panels). MKP-3 153–381 could not activate transcription on the PEPCK promoter (Figure 1B). Neither MKP-3 153–381 nor MKP-3 C293S has the capability to promote transcription of the G6Pase promoter (Figure 1B). It has been previously demonstrated that MKP-3 dephosphorylates FOXO1 on Ser256 and promotes nuclear translocation of FOXO1 [18], which subsequentially binds to the promoters of gluconeogenic genes and turns on the gluconeogenic program. Consistent with the results on gene transcription, both MKP-3 153–381 and MKP-3 C293S lost the ability to promote FOXO1 nuclear translocation in either vehicle treated or dexamethasone treated Fao cells (Figure 1C and figure S1). Examination of endogenous G6Pase gene expression also confirmed that neither MKP-3 153–381 nor MKP-3 C293S was able to increase expression of endogenous G6Pase gene (0.88±0.12 and 0.98±0.17, respectively, vs. GFP 1±0.38) in Fao cells while MKP-3 and MKP-3 S159AS197A significantly increased expression of G6Pase gene (3.42±0.62 and 5.27±1.35 respectively, Figure 2A). Similar results were observed with glucose output in Fao cells. MKP-3 153–381 and MKP-3 C293S were not able to increase glucose output compared to GFP control (12.05±0.182 and 12.29±0.25, respectively, vs. 13.48±0.58 μM/mg protein) while MKP-3 and MKP-3 S159AS197A significantly increased glucose output by 31.3% and 21% respectively compared to GFP control (Figure 2B).

### The domains mediating MKP-3 and FOXO1 interaction

**Figure 3:**
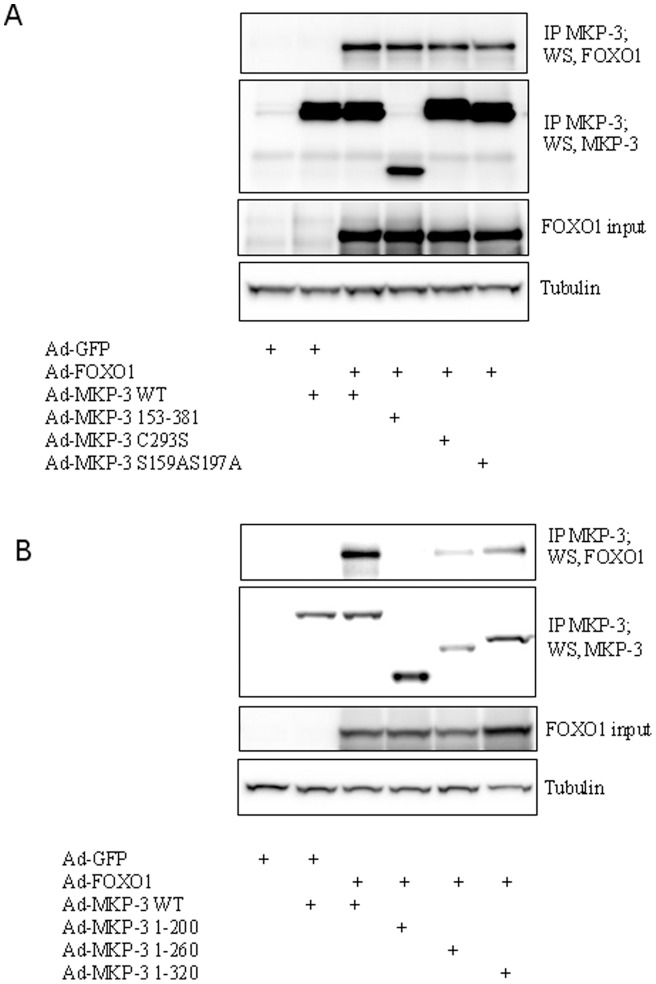
Mapping the functional domains of MKP-3 essential for mediating the interaction between MKP-3 and FOXO1. **A.** Co-immunoprecipitation of FOXO1 WT and MKP-3 WT or mutants deficient in ERK binding domain, deficient in phosphatase activity or deficient in ERK phosphorylation sites from Fao cells. **B.** Co-immunoprecipitaion of FOXO1 WT and MKP-3 WT or mutants truncated from C terminus.

To determine the relationship between MKP-3/FOXO1 interaction and the ability of MKP-3 to promote transcription of gluconeogenic genes, co-immunoprecipitation experiment was performed by co-expressing MKP-3 wild type (WT), MKP-3 153–381, MKP-3 C293S, or MKP-3 S159AS197A with FOXO1 WT. Despite the fact that MKP-3 153–381 and MKP-3 C293S are unable to promote gluconeogenesis, these two mutants can still interact with FOXO1, indicating that the activity of MKP-3 is not required for MKP-3/FOXO1 interaction (Figure 3A) and the c-terminal portion of MKP-3 is necessary for mediating MKP-3/FOXO1 interaction. Next, a serial of MKP-3 mutants with deletions in the c-terminus, including MKP-3 1–200, MKP-3 1–260, and MKP-3 1–320, were made and co-expressed with FOXO1 WT in Fao cells. The interaction between MKP-3 1–260 or MKP-3 1–320 and FOXO1 was still detectable (Figure 3B). In contrast, MKP-3 1–200 can not be co-immunoprecipitated with FOXO1 (Figure 3B). These results demonstrate that residues 200–260 are important for MKP-3/FOXO1 interaction.

**Figure 4:**
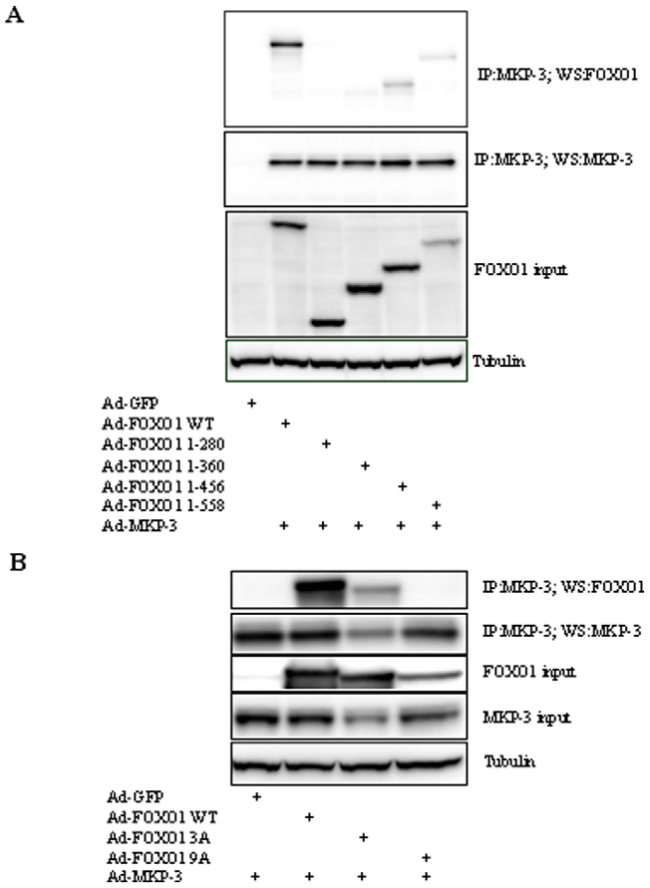
Mapping the functional domains of FOXO1 essential for mediating the interaction between MKP-3 and FOXO1. **A.** Co-immunoprecipitaion of FOXO1 WT or mutants truncated from C terminus and MKP-3 WT from Fao cells. FOXO1 WT or mutants and MKP-3 WT were co-expressed in Fao cells via adenovirus-mediated gene transfer. GFP was used as a control. **B.** Co-immunoprecipitaion of FOXO1 WT or mutants deficient in Akt or ERK phosphorylation sites and MKP-3 WT.

To analyze the domains of FOXO1 that are important for MKP-3/FOXO1 interaction, a serial of Flag-tagged FOXO1 mutants with deletions in the C terminus, including FOXO1 1–558, FOXO1 1–456, FOXO1 1–360 and FOXO1 1–280, were made and co-expressed with MKP-3 WT in Fao cells. FOXO1 1–558 has a much weaker interaction with MKP-3 compared to WT, indicating that residues 558–652 could be potentially important but the conclusion was complicated by the fact that protein input for FOXO1 1–558 was also lower. Truncation to residue 456 did not further weaken interaction with MKP-3 while FOXO1 1–360 and FOXO1 1–280 completely lost the interaction (Figure 4A), indicating that residues 360–456 are important for MKP-3/FOXO1 interaction. FOXO1 can be phosphorylated by Akt on three Ser/Thr residues (Thr24, Ser256, Ser319) and by ERK on nine Ser residues (Ser246, Ser284, Ser295, Ser326, Ser413, Ser415, Ser429, Ser467, Ser475). There is no overlap between Akt and ERK phosphorylated residues. The interaction between Akt phosphorylation resistant FOXO1 mutant, FOXO1 3A (all three Akt phosphorylation sites were mutated to alanine), and MKP-3, or between ERK phosphorylation resistant FOXO1 mutant, FOXO1 9A (all nine ERK phosphorylation sites mutated to alanine), and MKP-3, was also examined. Interestingly, FOXO1 3A still interacts with ERK but FOXO1 9A completely lost the interaction (Figure 4B).

### FOXO1 3A/9A and gluconeogenesis

**Figure 5:**
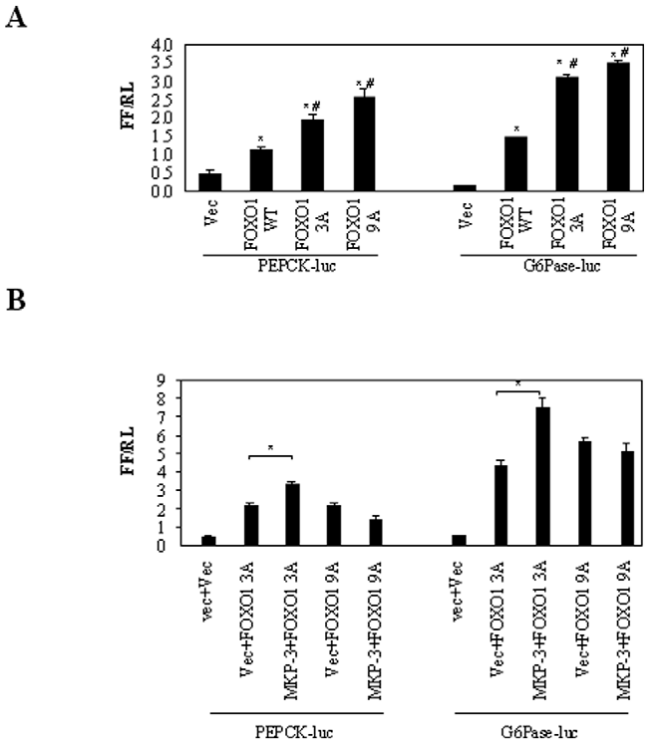
Akt/ERK phosphorylation resisitant FOXO1 mutants and MKP-3 promoted transcription of gluconeogenic genes. **A.** Effects of FOXO1 3A and 9A on transcription of PEPCK and G6Pase promoters. *, P<0.05 compared to vector control; #, P<0.05 compared to FOXO1 WT. **B.** Effects of FOXO1 3A and 9A on transcription of PEPCK and G6Pase promoters in the presence of MKP-3.

It is well established that Akt phosphorylation resistant FOXO1 3A has a stronger capability in inducing gluconeogenesis than wild type FOXO1. ERK activation can also decrease transcription of gluconeogenic gene [28]. However, the biological effect(s) of ERK phosphorylation on FOXO1 is not known. The effect of ERK phosphorylation resistant mutant, FOXO1 9A, on transcription of PEPCK and G6Pase promoters was studied. As shown in figure 5A, FOXO1 9A significantly increased transcription of both PEPCK and G6Pase promoters compared to wild type FOXO1 (221% and 240% of wild type FOXO1, respectively) and the effect is similar to that of FOXO1 3A (166% and 213% of wild type FOXO1, respectively), indicating that ERK phosphorylation on FOXO1 may potentially decrease FOXO1's gluconeogenic potency.

Our previous report demonstrated that MKP-3 has an additive effect with wild type FOXO1 on transcription of PEPCK and G6Pase promoters [18]. We also reported that MKP-3 can activate FOXO1 by at least dephosphorylating Ser 256, one of the Akt phosphorylation sites [18]. Interestingly, MKP-3 still has a significant additive effect with FOXO1 3A on transcription of PEPCK and G6Pase promoters (56% and 74% respectively) though the effect was smaller than that with wild type FOXO1 as we previously reported (Figure 5B). These results indicate that MKP-3 may dephosphorylate additional residue(s) in FOXO1 or indirectly affect FOXO1 3A by targeting other proteins, which will be interesting to investigate in the future. Surprisingly, MKP-3 does not have any additive effect with FOXO1 9A on transcription of PEPCK and G6Pase promoters (Figure 5B) though Ser 256 is still intact. This is possibly due to the fact that MKP-3 does not interact with FOXO1 9A, therefore does not have the access to Ser 256.

### FOXO1 3A rescues the hypoglycemia effect caused by MKP-3 knockdown in the liver of lean mice

**Figure 6:**
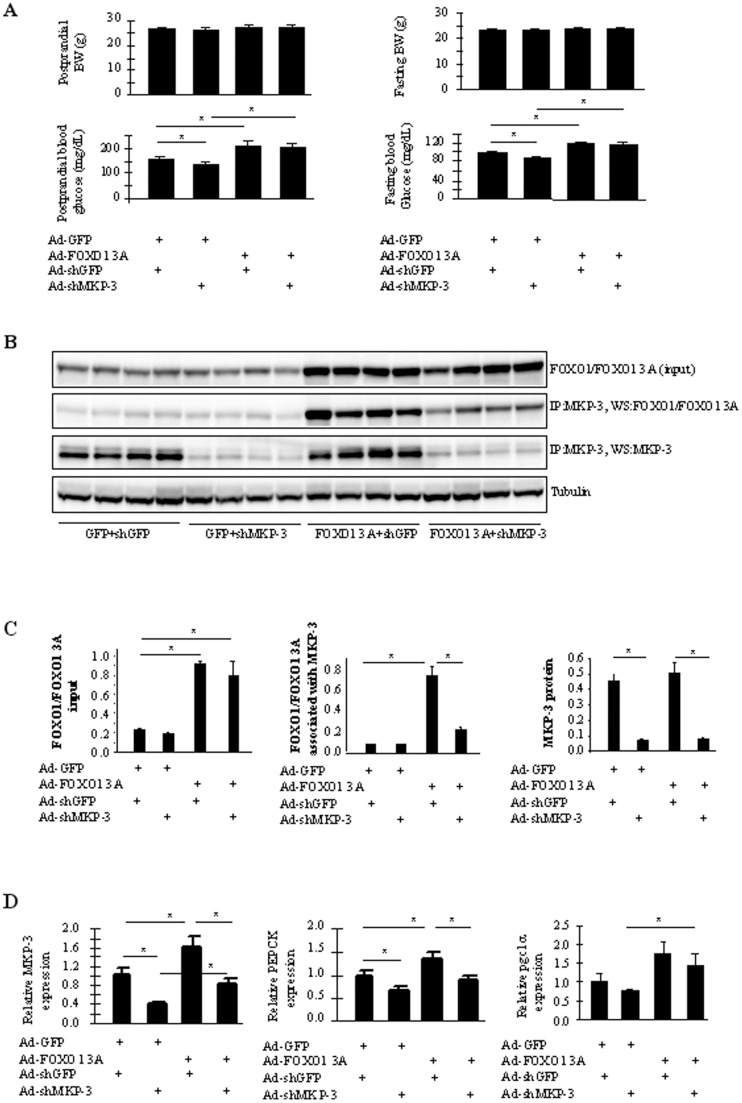
The Akt phosphorylation resistant FOXO1 mutant (FOXO1 3A) rescues the hypoglycemic effect caused by reduced MKP-3 expression in the liver of lean mice. **A.** Body weight and glucose levels in mice injected with adenovirus expressing shGFP, shMKP-3, GFP or FOXO1 3A (n = 8 for each froup). **B.** Protein expression of MKP-3 and FOXO1 in mice as described in A (n = 4 for each group). **C.** Quantitative graphs for B. **D.** Expression of gluconeogenic genes in mice as described in A (n = 8 for each group). *, P<0.05, between groups as indicated on graphs.

Our previous in vitro experiments in primary hepatocytes support the hypothesis that FOXO1 is a downstream target of MKP-3 since blocking the function of FOXO1 blunts MKP-3 promoted gluconeogenesis. In this study, in vivo experiments were performed to examine whether Akt phosphorylation resistant FOXO1 3A mutant can rescue the hypoglycemic effect caused by reduced hepatic MKP-3 expression. Although MKP-3 was only reported to dephosphorylate Ser256, phosphorylation of Ser256 is important for retaining FOXO1 in the cytosol by insulin and is also required for Thr24 and Ser319 to be phosphorylated [30]. Therefore FOXO1 3A is expected to function similarly to FOXO1 mutant containing S256A alone. Male C57BL/6J mice were separated into four experimental groups: the first group of mice were injected with adenovirus expressing GFP and adenovirus expressing a short hairpin interfering RNA against GFP (shGFP); the second group of mice were injected with adenovirus expressing GFP and adenovirus expressing shMKP-3; the third group of mice were injected with adenovirus expressing FOXO1 3A and adenovirus expressing shGFP; the fourth group of mice were injected with adenovirus expressing FOXO1 3A and adenovirus expressing shMKP-3. Knocking down MKP-3 in the liver of lean mice significantly reduced fed blood glucose levels by 13.2% and fasting blood glucose levels by 8.6% without affecting body weight (Figure 6A, first two bars in all graphs). Mice injected with adenovirus expressing FOXO1 3A and shGFP have significantly elevated blood glucose levels compared to mice injected with adenovirus expressing GFP and shGFP (30.5% and 21%, respectively, in fed and fasted state). Over-expression of FOXO1 3A also significantly increased blood glucose levels in mice co-injected with adenovirus expressing shMKP-3 compared to over-expression of GFP in mice co-injected with adenovirus expressing shMKP-3 (Figure 6A, 48% and 29.8%, respectively, in fed and fasted state). Western blot analysis confirmed over-expression of FOXO1 3A (294% and 241%, respectively) in the liver of mice administered with adenoviruses expressing FOXO1 3A and shGFP or shMKP-3 (Figure 6B, the first panel; Figure 6C, the first graph) compared to the first group of mice injected with Ad-GFP and Ad-shGFP. Reduced MKP-3 protein expression was also confirmed in mice injected with Ad-shMKP-3 and Ad-GFP or Ad-FOXO1 3A (83% and 81% respectively) compared to the first group of mice injected with Ad-GFP and Ad-shGFP (Figure 6B, the third panel; Figure 6C, the third graph). Consistent with our previous report and current study that MKP-3 and FOXO1 interacts and can be co-immunoprecipitated in Fao hepatoma cells [18], MKP-3 and FOXO1/FOXO1 3A can also be co-immunoprecipitated from liver lysates (Figure 6B, the second panel; Figure 6C, the second graph). Overexpression of FOXO1 3A led to an increase of 7.9 folds of FOXO1/FOXO1 3A protein to be associated with MKP-3 compared to GFP control (Figure 6C, the second graph, bar 3 vs. 1). Reduced hepatic MKP-3 expression by shMKP-3 decreased 71% of FOXO1/FOXO1 3A to co-immunoprecipitate with MKP-3 in FOXO1 3A overexpression mice (Figure 6C, the second graph, bar 4 vs. 3). Gene expression analysis showed that FOXO1 3A significantly increased expression level of both MKP-3 and PGC1α genes in mice injected with adenovirus expressing shMKP-3 compared to GFP control (Figure 6D). These results indicate that FOXO1 3A rescues the hypoglycemic effect caused by knocking down MKP-3 in the liver.

## Discussion

MAP kinase phosphatases are traditionally thought to attenuate the mitogenic pathway of MAP kinase signaling. Recent studies have provided strong evidence to show that MAP kinases play important roles in various aspects of metabolism as well. For example, JNK has been demonstrated to be an inflammatory kinase involved in the development of obesity related insulin resistance [31]; P38 has been shown to promote gluconeogenesis by direct phosphorylation of PGC-1α, therefore stimulating its transcriptional activity toward gluconeogenic genes [32], [33], [34]; activation of the MEK/ERK pathway can partially inhibit PEPCK expression and is sufficient to mediate the suppression of G6Pase gene expression by phorbol ester [27], [28]. Correspondingly, more evidence has surfaced to show that MKP kinase phosphatases also play important roles in regulating metabolism. MKP-2 has been shown to repress gluconeogenesis by dephosphorylation of P38 in hepatoma cells [35]; MKP-4 has been demonstrated to impair insulin-stimulated glucose uptake in 3T3-L1 adipocytes [36]; and the prototype dual specificity phosphatase MKP-1 was shown to play a role in the development of diet-induced obesity [37]. Recently, several dual specificity MAP kinase phosphatases, such as MKP-1, PAC-1 and MKP-5, have been demonstrated to play important roles in regulating innate immunity [19]. The rapid growing body of evidence support that MAP kinase phosphatases have a broader spectrum of functions than traditionally thought.

One important contributing factor to obesity related hyperglycemia is increased hepatic glucose output. Contrary to the role of MKP-2, which inhibits gluconeogenic gene expression and glucose output, our previous studies identified MKP-3 as the first dual specificity phosphatase that promotes gluconeogenic gene expression and glucose output in obese and insulin resistant state [17], [18]. The gluconeogenic capacity of MKP-3 is at least partially fulfilled through dephosphorylation of Ser256 of FOXO1 in the presence of insulin, thereby promotes its nuclear translocation and binding to the promoters of gluconeogenic genes. It remains to be studied whether MKP-3 can dephosphorylate additional residues in FOXO1 independent of insulin. Interestingly, it has been recently shown that PP2A can also dephosphorylate FOXO1 on Thr24 and Ser256 to regulate its nuclear import in islet beta cells in response to oxidative stress [38], revealing that FOXO1 dephosphorylation can be controlled by multiple phosphatases. The purpose of this study is to elucidate the mechanism of MKP-3/FOXO1 interaction and the effect on glucose output in vitro as well as FOXO1 as a downstream effecter of MKP-3 in vivo. The results from this study showed that the phosphatase activity of MKP-3 is essential for promoting transcription of gluconeogenic genes and glucose output though it is not required for MKP-3 to bind to FOXO1. By constructing MKP-3 and FOXO1 deletion mutants, the residues critical for mediating MKP-3/FOXO1 interaction have been identified.

The effects of Akt and ERK phosphorylation on MKP-3/FOXO1 interaction have also been examined by using Akt and ERK phosphorylation resistant FOXO1 3A and 9A mutants, respectively. MKP-3 still interacts with FOXO1 3A but surprisingly lost contact with FOXO1 9A. Interestingly, MKP-3 still has a small but significant additive effect with FOXO1 3A on transcription of PEPCK and G6Pase promoters, indicating potential dephosphorylation by MKP-3 on FOXO1 outside Akt phosphorylation sites. It has been reported that activation of the MEK/ERK pathway can also inhibit expression of PEPCK and G6Pase genes, most likely through a mechanism independent of insulin. Consistently, increased basal ERK activity and decreased expression of PGC1α and PEPCK genes have been observed in the liver of mice deficient in the signaling adapter p62, providing in vivo evidence that ERK activation is associated with repression of gluconeogenic gene expression [39]. It has been shown that ERK can phosphorylate FOXO1 on nine Ser residues, which are completely different from the three residues that can be phosphorylated by Akt [40]. However, the physiological significance of ERK mediated phosphorylation on FOXO1 is unclear. In this study, ERK phosphorylation resistant FOXO1 9A mutant has been demonstrated to strongly activate transcription of PEPCK and G6Pase promoters, supporting a role of ERK in suppressing gluconeogenesis. Further studies would be needed to clarify the role of ERK in repressing gluconeogenesis in vivo and whether it is subject to regulation by insulin since in vitro studies demonstrated that ERK is not required to mediate the effect of insulin on repressing expression of gluconeogenic genes. Interestingly, mutation of all nine ERK phosphorylatable residues in FOXO1 to alanine completely ablated the affinity of FOXO1 to bind to MKP-3. This may explain the fact that MKP-3 does not have any additive effect with FOXO1 9A on increasing transcription of PEPCK and G6Pase promoters though Ser256 is still intact in FOXO1 9A mutant. It is unclear how these nine mutations disrupted MKP-3/FOXO1 interaction but one explanation could be that FOXO1 protein structure is significantly altered due to reduced negative charges. Due to the fact that MKP-3 no longer interacts with FOXO1 9A, only FOXO1 3A was used in vivo to test whether over-expression of FOXO1 3A can rescue the hypoglycemic phenotype in hepatic MKP-3 knockdown mice. FOXO1 3A indeed significantly elevated blood glucose levels despite reduced hepatic MKP-3 expression. Results from this study support that FOXO1 is a substrate of MKP-3 in vivo.

## Supporting Information

Figure S1 **Localization of GFP-FOXO1 in Fao cells expressing a control protein, or MKP-3, or MKP-3 153-381, MKP-3 C293S, or MKP-3 S159AS197A. A.** Vehicle treated cells. **B.** Dexamethasone treated cells.
(TIF)
