# Supplementary figures and images for: Mapping MKP-3/FOXO1 Interaction and Evaluating the Effect on Gluconeogenesis
Source: PLoS One. 2012 Jul 25;7(7):e41168. doi: 10.1371/journal.pone.0041168 (PMC3405053; doi:10.1371/journal.pone.0041168)

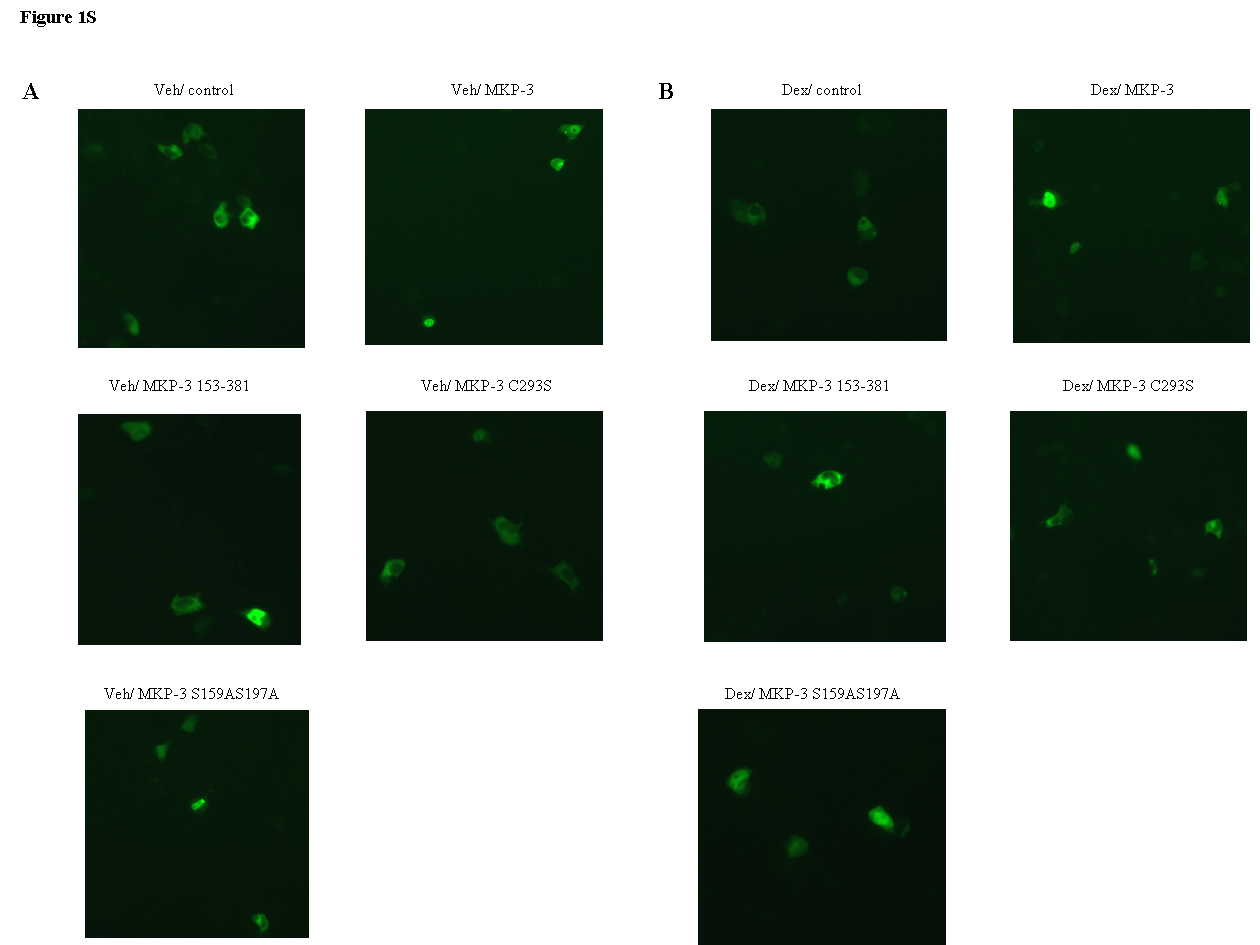

Supplement: Figure S1 — Localization of GFP-FOXO1 in Fao cells expressing a control protein, or MKP-3, or MKP-3 153-381, MKP-3 C293S, or MKP-3 S159AS197A. A. Vehicle treated cells. B. Dexamethasone treated cells. (TIF) [file pone.0041168.s001.tif]
